# Impact of the repurposed drug thonzonium bromide on host oral-gut microbiomes

**DOI:** 10.1038/s41522-020-00181-5

**Published:** 2021-01-22

**Authors:** Aurea Simon-Soro, Dongyeop Kim, Yong Li, Yuan Liu, Tatsuro Ito, Kenneth R. Sims, Danielle S. W. Benoit, Kyle Bittinger, Hyun Koo

**Affiliations:** 1 Biofilm Research Labs, Levy Center for Oral Health, School of Dental Medicine, University of Pennsylvania, Philadelphia, PA 19104 USA; 2 Department of Orthodontics and divisions of Pediatric Dentistry & Community Oral Health, School of Dental Medicine, University of Pennsylvania, Philadelphia, PA 19104 USA; 3 Department of Pediatric Dentistry, Nihon University School of Dentistry at Matsudo, Chiba, Japan; 4 Translational Biomedical Science Program, University of Rochester School of Medicine and Dentistry, Rochester, New York, NY 14642 USA; 5 Department of Biomedical Engineering, Department of Chemical Engineering, Materials Science Program, University of Rochester, Rochester, New York, NY 14627 USA; 6 Division of Gastroenterology, Hepatology, and Nutrition, The Children’s Hospital of Philadelphia, Philadelphia, PA 19104 USA; 7 Center for Innovation & Precision Dentistry, School of Dental Medicine, School of Engineering and Applied Sciences, University of Pennsylvania, Philadelphia, PA 19104 USA

**Keywords:** Microbiota, Microbiome, Plaque

## Abstract

Drug repurposing is a feasible strategy for the development of novel therapeutic applications. However, its potential use for oral treatments and impact on host microbiota remain underexplored. Here, we assessed the influences of topical oral applications of a repurposed FDA-approved drug, thonzonium bromide, on gastrointestinal microbiomes and host tissues in a rat model of dental caries designed to reduce cross-contamination associated with coprophagy. Using this model, we recapitulated the body site microbiota that mirrored the human microbiome profile. Oral microbiota was perturbed by the treatments with specific disruption of *Rothia* and *Veillonella* without affecting the global composition of the fecal microbiome. However, disturbances in the oral-gut microbial interactions were identified using nestedness and machine learning, showing increased sharing of oral taxon *Sutterella* in the gut microbiota. Host-tissue analyses revealed caries reduction on teeth by thonzonium bromide without cytotoxic effects, indicating bioactivity and biocompatibility when used orally. Altogether, we demonstrate how an oral treatment using a repurposed drug causes localized microbial disturbances and therapeutic effects while promoting turnover of specific oral species in the lower gut in vivo.

## Introduction

Topical antimicrobials from traditional medicines and natural products to synthetic agents have been extensively investigated for oral infection control but few have demonstrated effectiveness in vivo or have reached clinical trials. The development of new drugs is a long and costly process involving multiple studies for effectiveness and toxicity using both laboratory and in vivo models prior to clinical assessment of safety, therapeutic efficacy and potency^1^. Recently, the advent of drug repurposing has provided a pathway for previously FDA-approved drugs to find new clinical applications, such as for antimicrobial therapies. Advantages, including reduced development time and cost, have motivated the scientific community to apply repurposing strategies^1^ to find novel therapeutic applications for human use^2,3^. Recently, thonzonium bromide (TB), a monocationic detergent used for ear infection treatment, has been tested as a repurposed antibacterial agent in vitro^4,5^. Moreover, nanoparticle carriers (NPCs) have been developed to enhance TB delivery against oral biofilms using laboratory models^5,6^. However, its antimicrobial effects when topically delivered in the oral cavity as well as its impact on host tissues and microbiota under in vivo conditions are unknown, which could inform the potential bioactivity as well as disturbances of the repurposed drug treatment in the host.

Rodent animal models are widely used to study microbiomes applied to human health and disease^7,8^. Murine models using mice and rats have shown that disruption of the oral microbiome by opportunistic pathogens may be associated with oral diseases^9^. Recent studies demonstrated oral-gut microbiota connections, including the observation that orally derived bacteria could colonize and persist in the gut with host implications at the distant gastrointestinal site^10,11^. Despite differences between the human and rodent microbial composition, animal models can provide valuable information about microbiota disturbances across the gastrointestinal tract^12,13^. However, a major limitation is cross-contamination by coprophagy (i.e., ingestion of fecal material) making simultaneous evaluation of microbiota at different body sites challenging^14^. Hence, investigations to assess the impact of exogenous perturbations, such as topical antimicrobial treatments, on the microbiota across different body sites within the same animal remain sparse.

Microbiome structure and composition are niche dependent. Host, environment, and microbial interactions can define the microbial community within a particular body site^15–20^. Nested bacteria linked to a particular niche are called specialist bacteria whereas generalist bacteria are found in multiple niches^18,19,21,22^. Different sites throughout the gastrointestinal tract have distinctive microbial populations^23^. Stability of a particular microbial community depends on environmental factors including diet, host characteristics, or exogenous intervention, such as antimicrobial treatment^24,25^. Disturbances can locally affect the intervention site (i.e., where the antimicrobial is deployed) and adjacent areas^26,27^. Better knowledge of microbial ecology, as well as the interplay between local and distant microbiota, are key for understanding host-microbe interactions and development of more targeted antimicrobial therapies. Given that the gastrointestinal tract is a natural connection between oral and gut-specific microbiomes^23^, topical applications of TB could have a niche-specific effect. However, limited knowledge exists about whether topical oral antimicrobials can cause local and distant microbial disturbances across the gastrointestinal tract.

Here, we investigated the impact of TB, as a repurposed drug for oral topical treatment, on gastrointestinal microbiomes and host tissues using a rodent model of dental caries designed to prevent coprophagy. Our data revealed localized reduction of caries development on teeth without deleterious effects to oral mucosal and intestinal tissues, whereas niche-specific microbiota disturbances were observed following topical TB applications. Using a combination of microbiome sequencing and computational analyses, we found specific disruptions of *Rothia* and *Veillonella* (associated with dental caries) in the oral site but the global composition of the gut microbiome was unaffected by the treatment. However, specific alterations in the oral-gut microbial interactions were identified using nestedness and machine-learning approaches, showing increased sharing of oral taxon *Sutterella* in the gut microbiota. Altogether, we report how an oral treatment using a repurposed drug causes specific microbial changes and therapeutic effects in a feasible animal model for oral-gut microbiome assessment.

## Results

### Impact of repurposed drug topical treatment on host tissues and microbiomes in vivo

**Fig. 1:**
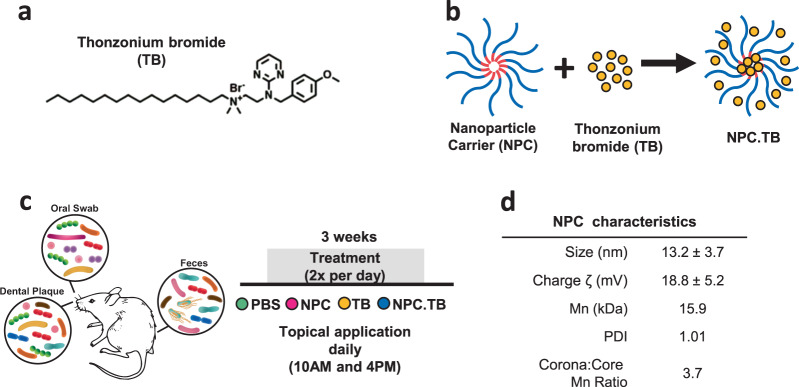
Animal model and treatment regimen for drug repurposing evaluation. **a** Thonzonium bromide (TB) chemical structure. **b** Cartoon illustration of TB-loaded into nanoparticle carrier. **c** Diagram of the animal experiment indicating the body site microbiome including oral (dental plaque and oral swab) and gut (feces). The treatment was performed twice a day for 3 weeks. Treatment groups included PBS, NPC, TB, and NPC.TB. **d** Table summarizing the characterization parameters of the NPC used in this study (NP13/3). PBS phosphate buffer saline, NPC nanoparticle carrier, TB thonzonium bromide, NPC.TB TB-loaded nanoparticle carrier, Mn number average molecular weight, PDI Polydispersity Index.

We used the rodent caries model to interrogate the effects of thonzonium bromide (TB) treatment on the host dental and soft tissues in the oral cavity as well as on the local and distant microbiota. TB (see Fig. 1a for chemical structure) is commonly used as a monocationic detergent to penetrate cellular debris during ear infection treatment and has been shown to have antimicrobial activity^28,29^. To test the effects of the repurposed drug, we used either TB free in solution or loaded into a nanoparticle carrier (NPC) designed to enhance drug delivery (Fig. 1b, d). We simulated treatment conditions that might be experienced clinically in humans by applying the solutions topically twice daily with a brief, 30 s exposure time. At the end of the experimental period, samples from swabbing the entire oral surfaces (oral swab), specific oral site (dental plaque on teeth), and distant gastrointestinal sample representing gut microbiota (feces) were used for microbiome analysis. In addition, oral soft tissues, teeth, and organs were analyzed (Fig. 1c). For dental (tooth) tissues, we found that the repurposing drug TB (TB and NPC.TB) reduced the number of severe lesions compared to control (PBS) group but did not prevent disease onset (*P* < 0.05; Supplementary Fig. 1a). However, viable colonies of caries-related *Streptococcus* and *Candida* in dental plaque showed no significant differences among treatment groups (Supplementary Fig. 1b), suggesting that the anticaries effect may be related to other microbial components associated with the disease. Analysis of the soft oral and small/large intestine tissues by hematoxylin and eosin staining revealed no differences between TB treatments and controls, whereas no visible signs of adverse effects, such as proliferative changes, inflammatory responses, or necrosis were observed, consistent with lack of cytotoxic effects of this FDA-approved drug (REF. N050356 001) (Supplementary Fig. 1c). We next assessed the influences of TB applications on oral and gut microbiomes.

### Unique bacterial communities in rat and human body sites

**Fig. 2:**
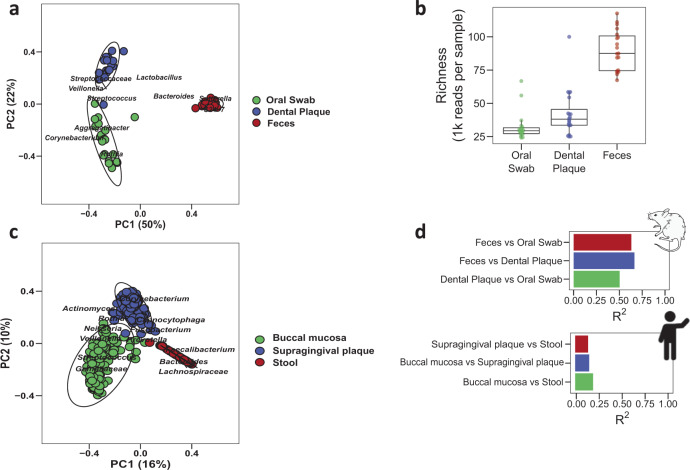
Unique bacterial communities in rat and human body sites. Sample types corresponding to oral (oral swab and dental plaque) and gut (feces) represented as body sites. **a** Bray–Curtis distances principal coordinate analysis for rodent samples. Each point corresponds to a sample colored by body site. Circle shapes show 95% confidence of the group. **b** Alpha diversity as richness showed statistical differences between sample types (*P* < 0.001, Wilcoxon test). **c** Beta-diversity using Bray–Curtis distances for the Human Microbiome Project samples showed differences between body sites. **d** Rat and human body site differences using pairwise PERMANOVA test for Bray–Curtis distances.

We first assessed the feasibility of the rodent model of dental caries to analyze the gastrointestinal microbiota at different sites. To evaluate niche-specific differences, we collected oral swabs, dental plaque, and feces from rats. In this model, the animals were housed in suspended wired cages designed to reduce coprophagy^30^, which can mislead body site microbiomes^14,31^. We found that the composition of the microbiota differed substantially between the three body sites (Fig. 2a), especially between fecal samples and the oral/plaque samples (*P* = 0.001, PERMANOVA test), suggesting no cross-contamination. Fecal samples had higher abundance of gut microbial taxa, such as *Bacteroides*, *Bacteroidales S24-7*, and *Sutterella*, relative to oral swab and dental plaque samples, which were dominated by *Streptococcus* genera (but not in fecal samples). Conversely, oral sample types (i.e., swabs and dental plaque) differed from each other (*P* = 0.001) to a lesser degree. Oral sites were distinguished primarily by higher abundance of *Rothia* in oral swab samples. The richness or number of species-level bacterial taxa also differed between body sites, with the fecal site containing more bacterial taxa than oral swabs (Fig. 2b, *P* = 0.001). Comparison of Shannon diversity and Faith’s phylogenetic diversity analysis of these samples yielded similar results (Supplementary Fig. 2a, b). Also, we observed oral treatment did not affect the niche-specific microbial community found in the oral-gut sites (Supplementary Fig. 2c). The results highlight the higher number and distinct composition of bacterial species in the lower gut, relative to oral sites.

To confirm whether the microbiota of our animal model reflected the same overall pattern found in the human body, we used 16S rRNA marker gene sequence data from the Human Microbiome Project to carry out a similar body site comparison. We selected buccal mucosa, supragingival plaque, and stool samples, then repeated our analysis from the rodent model (Fig. 2c). Consistent with the animal data, all three body sites were found to differ by beta-diversity analysis (*P* < 0.003, PERMANOVA pairwise comparisons). However, the degree of difference between body sites was greater in the rodent model than in humans (Fig. 2d). Thus, we show that our animal model recapitulates the microbiota separation between body sites found in humans, but with a greater degree of difference between sites.

### Effect of repurposed drug treatment on the microbiota across different gastrointestinal sites

**Fig. 3:**
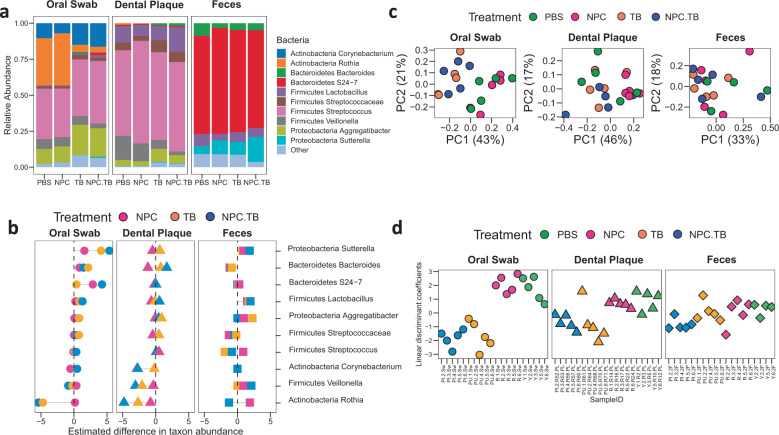
Effects of repurposed drug treatment on oral and gut microbiomes. **a** Bacteria relative abundances within rodent body sites for each treatment group. **b** Fold change estimate showing main bacterial genera that were differentially abundant between treatment groups against baseline (PBS). **c** Bray–Curtis principal coordinate analysis for each body site. Color samples correspond to treatment groups. **d** Linear discriminant coefficients obtained for treatment model. Higher coefficients indicate samples more likely to have non-TB treatments (PBS and NPC groups), while lower numbers indicate samples more likely treated with TB (NPC.TB and TB groups).

Using this model, we investigated the bacterial composition of oral-gut body sites in each treatment. Overall, oral sites were dominated by *Streptococcus* with means of 37% and 63% for oral and dental samples, respectively, whereas feces were predominated by *Bacteroidetes S24-7* with 70% relative abundance (Fig. 3a). Notably, we found dissimilar microbial proportions when comparing treatments within each sample type. Then, we asked whether treatment was affecting specific taxa in a particular body site through estimated differences in taxonomic abundances by comparing to PBS as a baseline group (Fig. 3b, Supplementary Fig. 2d). Using oral swabs, we found decreased abundance of *Rothia* (negative values) and increased abundance of *Sutterella* (positive values) in TB and NPC.TB groups. In dental plaque, *Veillonella* and *Rothia* were depleted in TB-containing treatments (TB and NPC.TB) but NPC alone was devoid of any disruptive effects (*P* < 0.05 for each taxon when compared to PBS). To assess the disturbances of the microbial community by treatment, we computed the Bray–Curtis distance between each pair of samples. Among oral swab samples, we found that the TB-containing groups were different from the groups that were not treated with TB (Fig. 3c, *P* = 0.007, PERMANOVA test).

Next, we used a Linear Discriminant Analysis (LDA) to assess whether the microbial disturbance was related to treatment and body site (Fig. 3d). Given that TB-containing groups (TB and NPC.TB) differed from non-TB-containing groups (PBS and NPC) in our analysis of community similarity, we grouped samples by TB exposure for LDA assessment. Lower linear discriminant coefficient scores indicated that the samples were more likely being treated with TB using microbiome by sample as a predictor. We obtained a linear discriminant coefficient separation between TB-containing groups in both oral sites but not in gut samples (*P* < 0.001, *P* < 0.01, and *P* > 0.05 for oral swab, dental plaque, and feces, respectively). Thus, bacterial taxa could discriminate between treatments in oral sites, but not in feces.

### Impact of repurposed drug on bacteria shared between body sites in a host

**Fig. 4:**
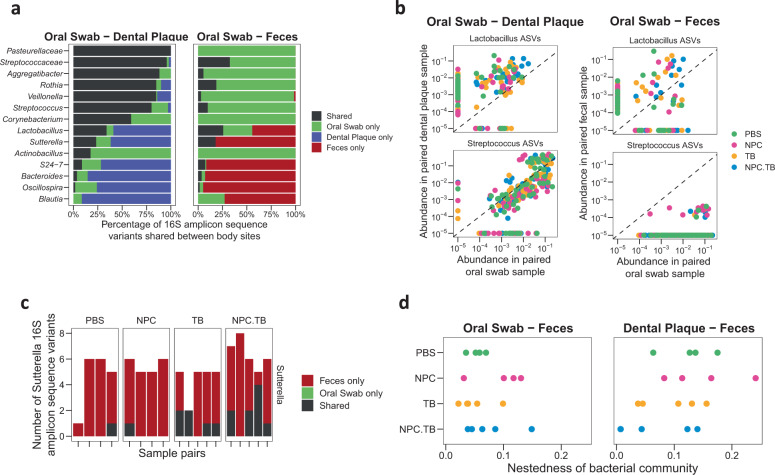
Impact of repurposed drug on bacteria shared between body sites in the host. **a** Common bacterial ASV between dental plaque and oral swab samples, and between oral swab and fecal samples. **b** *Streptococcus* and *Lactobacillus* ASVs shared between oral swab–dental plaque and oral swab-fecal paired samples. **c** Number of *Sutterella* ASVs shared in oral-fecal samples. Each column is a sample facet by treatment groups. **d** Nestedness, or shared bacterial community, found in each treatment group when compared between oral and gut sites. Each point corresponded to a sample. ASV amplicon sequence variant.

Having characterized the impact of drug repurposing on the average microbiota profile in local and distant body sites, we wanted to determine whether drug repurposing disrupted the levels of specific bacteria shared between body sites within a host. We analyzed the relative abundance of each bacterial 16S amplicon sequence variant (ASV) between body sites in each host. The ASV is the most fine-grained unit of taxonomic analysis possible with 16S rRNA marker gene sequencing. As expected, we found that more ASVs were shared between the oral swab and dental plaque, relative to oral and fecal sites (Fig. 4a). The proportion of shared ASVs differed by genus for oral swab–dental plaque (*P* < 0.001) and oral swab-fecal comparisons (*P* < 0.001). As illustrative examples, we consider *Streptococcus*, which is prominent in the oral microbiota but not in the gut, and *Lactobacillus*, which is present at both sites. The relative abundance of shared *Streptococcus* ASVs was higher in oral samples relative to dental plaque than that of *Lactobacillus* (Fig. 4b, left panel). Conversely, the abundance of shared *Lactobacillus* ASVs was higher in dental plaque, suggesting niche-specificity within oral sites.

In oral-gut comparison, fewer bacterial ASVs were shared between oral and fecal sites and the level of overlap was highly species dependent on genus (Fig. 4b, right panel). Only a small number of *Streptococcus* ASVs were shared between oral and fecal sample pairs, while a substantial fraction of *Lactobacillus* ASVs were found in both sites (Fig. 4b, Supplementary Fig. 4a). Thus, we found distinctive microbial community in oral-gut niches excepting specific bacteria taxa present along the gastrointestinal tract. We next asked whether drug repurposing altered the fraction of shared bacteria between local and distant body sites. For each genus, we carried out a logistic regression to determine if treatments with TB-containing groups or non-TB groups were associated with the fraction of oral ASVs appearing in oral swab or feces (Supplementary Fig. 3). We found that TB treatment was associated with an increased number of oral *Sutterella* ASVs detected in feces (*P* = 0.02, Fig. 4c, Supplementary Fig. 4c), indicating that TB treatments increased the level of shared bacteria between the oral cavity and gut.

Moving towards a broader picture, we investigated whether the total number of shared ASVs between sample types could be attributed to an environment-dependent pattern, such as host gastrointestinal sites. We felt that this possibility was important to consider due to the large difference in bacterial richness among oral, dental plaque, and fecal samples (Fig. 2b). The number of unshared species that can be attributed to a difference in diversity is characterized by the nestedness between samples. We computed the degree of nestedness between oral swab-fecal sample pairs, and again for dental plaque-fecal sample pairs (Fig. 4d; also see Methods for coding details). Analysis by linear models indicated that treatment with TB decreased the level of nestedness between plaque and fecal samples (*P* = 0.03), meaning that a smaller fraction of unshared species was attributable to a difference in particular body site diversity.

Thus, drug repurposing not only affected the average abundance of bacteria between body sites, but also the way that individual bacteria were shared between these host sites. Both analyses, the shared ASVs as single microbial taxon and the bacterial community nestedness, point toward increased sharing of bacterial ASVs between body sites following oral treatment with the repurposed drug TB.

## Discussion

In this study, we investigated the impact of a repurposed FDA-approved drug thonzonium bromide (TB) on niche-specific gastrointestinal microbiota and its therapeutic effect using an animal model of dental caries. We recapitulated the distinctive body site microbiota found in the human microbiome, indicating a feasible in vivo model to assess the influences of topical treatments on both oral-gut tissues and microbiomes. Signature oral bacteria present in oral sample types were *Streptococcus* and *Veillonella*, whereas *Lactobacillus* was shared between oral and gut body sites. The repurposed drug TB, used as a topical agent to control dental caries, showed specificity towards oral microbiome. We found specific depletion of the oral bacteria taxa *Rothia* in oral swab and reduction of both *Rothia* and *Veillonella* in plaque, but the gut microbiome was unaffected by TB treatments. *Rothia* and *Veillonella* has been associated with dental caries^32^, which could explain the reduction of severity of dental lesions observed in the animal model (Supplementary Fig. 1a). However, we found that TB treatment can influence the microbial interactions in the oral-gut axis by increasing the sharing of particular oral bacteria (i.e., *Sutterella*) between the body sites as determined using a machine-learning classifier and computational analyses. Altogether, we report selective antimicrobial modulation by topical oral treatments of a repurposed drug on repertoires of both local and distant microbiomes, while demonstrating therapeutic activity without cytotoxicity on host soft tissues.

Drug repurposing has been proposed as an alternative for new therapeutic applications using FDA-approved drugs^1^. Repurposed drugs have been explored as antimicrobial treatments to target pathogens^33–37^. Previously, either free or nanoparticle carrier (NPC) loaded TB was tested for repurposing as antimycotic and antibacterial agents, with promising in vitro data^19,30^. Here, we investigated the antimicrobial activity of TB in vivo. At the end of three weeks of treatment, we found reductions in the levels of *Rothia* and *Veillonella* in the oral sites, which have been associated with caries severity in children^32^. Conversely, TB treatments did not cause significant disturbances in the gut microbiota. The use of NPC did not significantly increase TB efficacy although it modulated sharper effects on *Veillonella* levels within plaque. The effects on oral microbiota by TB treatments were also reflected in host dental and soft tissues. The caries data show that TB/NPC.TB disrupted disease severity, indicating that specific alterations in the local oral microbiota provided detectable therapeutic effect in vivo. In contrast, oral mucosal and intestinal tissues were unaffected by TB, suggesting biocompatibility. Whether a more prolonged experimental period could lead to further disruption of the oral microbiome, caries development, or soft-tissue histopathology remain to be assessed in future studies.

Several studies have linked oral microbiome communities to gastrointestinal diseases such as inflammatory bowel disease and colorectal cancer^38,39^. Disruption of local microbiota might affect distant microbial communities within the gastrointestinal tract^40^. To identify how an orally applied drug might affect the microbiota among body sites, we used machine learning and nestedness analyses. Discriminate coefficients in oral sites showed microbial disturbances that were associated with repurposed drug treatment. Gut microbiota was not globally affected by oral applications of TB but it induced increased levels of oral *Sutterella* species^11,41,42^ in the gut microbiome. These findings suggest that the localized disturbances on oral microbiome by TB-containing treatments might promote turnover of specific species in distant gastrointestinal sites although its functional impact on the lower gut physiology needs to be further elucidated^43^. It is noteworthy that this oral-gut microbiota connection could not be identified using conventional methods for microbiome composition, indicating that inclusion of machine learning and nestedness analyses may provide a more integrated assessment of new drugs across the gastrointestinal tract.

**Fig. 5:**
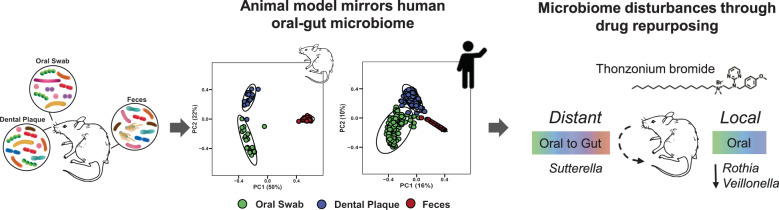
Oral-gut microbiome rodent model and microbiota disturbances by thonzonium bromide. Gastrointestinal microbiome differs between oral-gut body sites. Our rodent model showed similar pattern of the microbial communities in oral (oral swab and dental plaque) and gut (feces) sites in human reported previously. This model was then applied to assess the impact of thonzonium bromide in the host microbiota; topical oral treatment caused localized microbiome disturbances while promoting turnover of specific oral species in the gut microbiota.

In conclusion, we found how the oral and gut microbiota can be distinctively perturbed by an exogenous topical drug intervention whereby localized disruption of plaque microbiome composition was accompanied by increased sharing of specific oral species in the gut bacterial community. We also demonstrated that an FDA-approved drug, thonzonium bromide, has potential to be repurposed for topical oral treatment showing therapeutic (anticaries) properties without deleterious effects on both oral and intestinal tissues. Therapeutic activity along with multiorgan microbiome changes across the gastrointestinal tract can be analyzed using this model (Fig. 5). Assessment of changes in local (on-target) and distant (off-target) microbiome composition and ecological interactions after therapy may provide an enhanced understanding of the bioactivity and potential side-effects of current and novel therapeutic agents in an integrative manner. Future studies could investigate other important host factors including diet, host immunity, and environmental stress on the various microbiomes and the corresponding impacts on health and disease.

## Methods

### Rodent model of dental caries

The therapeutic efficacy and impact on the oral-gut microbiome of thonzonium bromide were assessed on a host-infection rat caries model^44–46^. Briefly, 48 Sprague Dawley SPF rats (specific pathogen-free) aged 15 days were purchased with their dams from Harlan Laboratories (Madison, WI). Upon arrival, animals were screened for *Streptococcus mutans* and *Candida albicans* and were determined not to be infected with these organisms using selective culture media as described previously^44,46^. The animals were then infected by mouth with actively growing (mid-logarithmic) culture of *S. mutans* serotype c (bacterial pathogen associated with human dental caries, OD600 = 1, 10^9^ CFUs/ml of *S. mutans* UA159) and *Candida albicans* (fungal organism associated with severe childhood caries, OD600 = 0.85, 10^7^ CFU/ml of *C*. *albicans* SC5314), and their co-infection were confirmed at 21 days via oral swabbing^44,46^. No antibiotics were used before infection. The animals were randomly placed into suspended cages by pairs according to treatment groups (see Topical oral treatment and experimental groups section), and then each of the agents applied topically in the mouth using a custom-made applicator^44^. Each group was provided the National Institutes of Health cariogenic diet 2000 (TestDiet, St. Louis, MO, USA) and 5% sucrose water ad libitum^30^. The experiments proceeded for 3 weeks. All animals were weighted weekly, and their physical appearance were noted daily. At the end of the experimental period, the animals were sacrificed and the microbiome as well as the host dental and soft tissues analyzed as described in the following sections.

### Ethics statement

The animal experiment was conducted in strict accordance with the guidelines of the Animal Welfare Act of the United States, under the protocol reviewed and approved by the Institutional Animal Care and Use Committee of the University of Pennsylvania (IACUC#805735 to H.K.).

### Polymeric nanoparticle carrier synthesis, characterization, and drug loading

We used thonzonium bromide (TB), either in solution or loaded into a polymeric nanoparticle carrier (NPC), as described previously^47^. Briefly, nanoparticles were formed of diblock copolymers synthesized in two steps, as described previously^47^. The cationic corona block of poly(dimethylaminoethyl methacrylate), or p(DMAEMA), was synthetized via reversible addition fragmentation chain transfer (RAFT) polymerizations using 40 wt% dimethylformamide as the solvent and 4-cyano-4- [(ethylsulfanylthiocarbonyl)sulfanyl]pentanoic acid (ECT) and 2,2-Azobisisobutyronitrile (AIBN) as the chain transfer agent (CTA) and initiator, respectively. The corona block reaction occurred at 60 °C for 6 h after a 45-min nitrogen purge. The reaction product was precipitated using pentane and diethyl ether before being dried under vacuum overnight. Then, the hydrophobic core block was synthesized via RAFT polymerization using p(DMAEMA) as the macroCTA and AIBN as the initiator to obtain poly(dimethylaminoethyl methacrylate)-b-poly(dimethylaminoethyl methacrylate-co-butyl methacrylate-co-propylacrylic acid), or p(DMAEMA)-b-p(DMAEMA-co- BMA-co-PAA). This reaction proceeded at 60 °C for 24 h after a 45-min nitrogen purge. Pentane and diethyl ether were used to precipitate the product for drying prior to further use. Raw polymer was dialyzed against water for purification (6–8 kDa membrane tubing) and lyophilized using a Labconco FreeZone 2.5 freeze dryer. Polymer molecular weights and polydispersities were determined using gel permeation chromatography (GPC, Shimadzu Technologies), including a miniDAWN TREOS multi-angle light scattering detector and Optilab T-rEX refractive index detector (Wyatt Technology) with DMF + 0.05 M LiCl as the mobile phase at 60 °C, as described previously^47^. Moreover, size and zeta potential of self-assembled NPCs following bath sonication were measured using a Zetasizer Nano ZS (Malvern Panalytical), as described previously^47^. Finally, nanoparticle carriers (NPC) were loaded with TB by combining preweighed lyophilized diblock copolymers and known volumes of concentrated thonzonium bromide solutions (in DMSO) with PBS in 20 mL glass scintillation vials. The resulting solutions were bath sonicated and stored at 4 °C until ready for use. Control groups, such as NPC alone and phosphate-buffered saline (PBS), were also prepared and tested.

### Topical oral treatment and experimental groups

All animals were randomly placed into treatment groups, and their teeth were treated topically twice daily. The topical oral treatment was performed using a custom-made applicator that allows application of defined volume (125 ± 25 µL) in the oral cavity^44^. The treatment groups were: (1) vehicle control (PBS), (2) NPC, (3) TB, and (4) NPC.TB. Each animal was treated at 10 am and 4 pm for 30 s to mimic daily use of oral therapeutics, and the treatment regimen was conducted for 3 weeks. TB concentration was 0.4 mg/ml (in phosphate buffer saline, or PBS), whereas the NPC concentration was 0.5 mg/ml (in PBS). NPC.TB treatment solution contained 0.4 mg TB/ml and 0.5 mg NPC/ml. The treatment was blinded by placing the test agents in color-coded vials. All animals were weighed weekly, and their physical appearances were noted daily until the end of the experimental period.

### Oral tissues and organs collection and analysis

At the end of the experimental period, the animals were sacrificed using standard procedures, and the jaws, oral tissues (tongue and mucosal tissues on the buccal side) and organs (small and large intestine) were surgically removed. All soft tissues and organs were kept in 10% formalin solution for histochemical staining. The jaws were aseptically dissected and prepared for dental plaque removal for microbiome analyses (see next section). All soft tissues/organs were harvested as part of the in vivo study and were kept in 10% Neutral Buffered Formalin (NBF) at 4 °C for at least 2 weeks until they were removed in preparation for Hematoxylin and Eosin (H&E) staining. Tissues were dissected, embedded in paraffin, cut into 4-μm sections, and mounted onto microscope slides for imaging. H&E staining was used to observe tissue morphology. Tissue dissection, processing, and H&E staining were performed under guidance by a lead technician within the Center for Musculoskeletal Research Histology, Biochemistry, and Molecular Imaging (HBMI) Core at the University of Rochester Medical Center. An Olympus VS120 Virtual Slide Microscope and VS-ASW FL software were used to obtain images of each tissue at 40× magnification. Representative sections of these images were selected and evaluated using QuPath open source version 0.1.2, quantitative pathology, and bioimage analysis software^48^. Histological changes were analyzed based on inflammatory signs, necrotic changes including necrosis, fibrosis, nuclear changes, abscesses, and cell degeneration. A surgeon pathologist within the Department of Pathology and Laboratory Medicine at the University of Rochester School of Medicine and Dentistry analyzed the histology in a blinded manner.

### Sample collection for microbiome sequencing

Oral swab was collected using moisten swab tip in microbial DNA-free water pretreated with UV cross-linker for 1 h before use to prevent DNA contamination (519 C FLOQSwab, COPAN). The sample was collected one day before the end of the experimental period (day 42). For each rat, the oral cavity was sampled for 30 s, rotating the swab on the entire oral surfaces, including checks, tongue, and teeth. Then, the swabs were placed back to the tube. Fecal samples were also collected by placing a fecal pellet in a sterile 1.5-ml tube. Since the animals were in suspended wired cages (Lenderking Caging Products, Millersville, MD), fresh fecal pellets were collected from the trays deposited below the cages using sterile inoculation loops. Then, the samples were placed in a sterile 1.5 ml tube and kept in −80 °C until sequencing processing.

The jaws were surgically removed and aseptically dissected after the animal being sacrificed to obtain dental plaque samples. The plaque was recovered from teeth by sonication in 5 ml of 154 mM sterile NaCl solution (three 10 s pulses with 30 s intervals at a watt output of ~7–8 W). The suspensions were serially diluted and plated on MSB or ChromAgar to estimate the *S. mutans* and *C. albicans* population, respectively. We stored 1 ml of plaque suspension for sequencing purposes. All samples were kept in −80 °C until DNA extraction. All jaws were defleshed and the teeth were prepared for dental caries scoring according to Larson’s modification of Keyes’ system^30^. A calibrated examiner who was blind for the study by using codified samples performed caries scoring.

Contamination control samples were included during the experimental process. We obtained samples during the animal experiments as blank oral swab and DNA-free water used for tip moisten. Also, we included experimental controls, including reagent controls, as negative controls, and mock DNA samples as positive controls following the reported methods^31^. We obtained a total of 80 samples divided into 60 experimental samples including feces, dental plaque, and oral swab (20 samples for each type), and 20 contamination controls.

### DNA extraction and amplification

Cells were pelleted from dental plaque, by centrifuging at maximum speed for 5 min. DNA was extracted from the pellets using the DNeasy PowerSoil kit (Qiagen, Valencia, CA, USA) according to the manufacturer’s instructions within a sterile class II laminar flow hood. Mock extractions were included to control for microbial DNA contamination arising through the sonication and extraction processes, respectively. PCR amplification of the V1–V2 region of the 16 S rRNA gene was performed using Golay-barcoded universal primers 27 F and 338 R. Four replicate PCR reactions were performed for each sample using Q5 Hot Start High Fidelity DNA Polymerase (New England BioLabs). Each PCR reaction contained: 4.3 µl microbial DNA-free water, 5 µl 5X buffer, 0.5 µl dNTPs (10 mM), 0.17 µl Q5 Hot Start Polymerase, 6.25 µl each primer (2 µM), and 2.5 µl DNA. PCR reactions with no added template or synthetic DNAs were performed as negative and positive controls, respectively. PCR amplification was done on a Mastercycler Nexus Gradient (Eppendorf) using the following conditions: DNA denaturation at 98 °C for 1 min, then 20 cycles of denaturation 98 °C for 10 s, annealing 56 °C for 20 s and extension 72 °C for 20 s, last extension was at 72 °C for 8 min. PCR replicates were pooled and then purified using a 1:1 ratio of Agencourt AMPure XP beads (Beckman Coulter, Indianapolis, IN), following the manufacturer’s protocol. The final library was prepared by pooling 10 µg of amplified DNA per sample. Those that did not arrive at the DNA concentration threshold (e.g., negative control samples) were incorporated into the final pool by adding 12 µl. The library was sequenced to obtain 2 × 250 bp paired-end reads using the MiSeq Illumina^49^.

### 16S rRNA analysis

To analyze 16S RNA gene sequences, we used QIIME2 v19.4^50^. We obtained taxonomic assignments based on the GreenGenes 16 S rRNA gene database v. 13_8^51^. We obtained an amplicon sequence variant (ASV) for analysis of shared and unique bacterial taxa in each body site through DADA2^52^. For human samples, sequences available at Human Microbiome Project database corresponding with 454 sequences region V1–V3 from buccal mucosa, supragingival plaque, and stool. Then, we trimmed the pyrosequences using Cutadapt^53^ and obtained ASV using plugging DADA2 denoise-pyro specifically designed for pyrosequencing sequences.

### Quantification and statistical analysis

We used the R environment for statistical computing version 3.4.4 for statistical analysis and data processing. The *p* value threshold used to ascertain statistical significance was *P* < 0.05. We used the Wilcoxon rank sum test to compare alpha diversity between body sites. The principal coordinate analysis for beta diversity was performed using the ape library for the R programming language^54^. To assess differences between groups based on Bray–Curtis distance, we used the PERMANOVA test^55^, as implemented in the vegan^56^ and pairwise Adonis^57^ libraries for R.

To assess differences in bacterial taxon relative abundances, we used a *t*-test of log-transformed relative abundance. We applied a false discovery rate (FDR) correction for multiple comparisons. Linear discriminant analysis was carried out using the lda function from MASS v.7.3.51.4^58^. Linear discriminant coefficients were tested within each body site using ANOVA with Bonferroni correction for multiple comparisons.

To analyze ASVs in paired samples, we tallied the number of ASVs present in one or both body sites. To test for independence between the number of shared ASVs and the genus assignment of the ASVs, we used a Chi-square test. To test for a difference in the number of shared ASVs within each genus based on treatment, we used a logistic mixed effects model with a random intercept for each mouse (oral swab–dental plaque) or cage (oral swab–feces). To compute the degree of nestedness and turnover between paired samples, we used the betapart library for R v1.5.1^59^. We compared the degree of nestedness after logit transformation using a linear model.

### Reporting summary

Further information on research design is available in the Nature Research Reporting Summary linked to this article.

## Supplementary information

Supplementary Information

Reporting Summary

## Data Availability

Sequences are deposited in the public repository NCBI Sequence Read Archive (SRA) accession number (SRP290845).

## Code availability

The code is available in GitHub (https://github.com/aureasimon/Body-sites).
