# Supplementary material for: Impact of the repurposed drug thonzonium bromide on host oral-gut microbiomes
Source: NPJ Biofilms Microbiomes. 2021 Jan 22;7:7. doi: 10.1038/s41522-020-00181-5 (PMC7822857; doi:10.1038/s41522-020-00181-5)
Supplement: Supplementary file 1 — Supplementary Information [file 41522_2020_181_MOESM1_ESM.pdf]

## Supplementary information

# Impact of the repurposed drug thonzonium bromide on host oral-gut microbiomes

Aurea Simon-Soro[1,2], Dongyeop Kim[1,2], Yong Li[1,2], Yuan Liu[1,2], Tatsuro Ito[1,2,3], Kenneth R. Sims Jr.[4], Danielle S. W. Benoit[4,5], Kyle Bittinger[6]*, Hyun Koo[1,2,7]*

[1]Biofilm Research Labs, Levy Center for Oral Health, School of Dental Medicine, University of Pennsylvania, Philadelphia, 19104, PA, USA.
[2]Department of Orthodontics and divisions of pediatric Dentistry & Community Oral Health, School of Dental Medicine, University of Pennsylvania, Philadelphia, 19104, PA, USA.
[3]Department of Pediatric Dentistry, Nihon University School of Dentistry at Matsudo, Chiba, Japan.
[4]Translational Biomedical Science Program, University of Rochester School of Medicine and Dentistry, Rochester, New York 14642, USA.
[5]Department of Biomedical Engineering, Department of Chemical Engineering, Materials Science Program, University of Rochester, Rochester, New York 14627, USA.
[6]Division of Gastroenterology, Hepatology, and Nutrition, The Children's Hospital of Philadelphia, Philadelphia, PA 19104, USA.
[7]Center for Innovation & Precision Dentistry, School of Dental Medicine, School of Engineering and Applied Sciences, University of Pennsylvania, Philadelphia, 19104, PA, USA.

*Correspondence: bittingerk@email.chop.edu (Kyle Bittinger), koohy@upenn.edu (Hyun Koo)

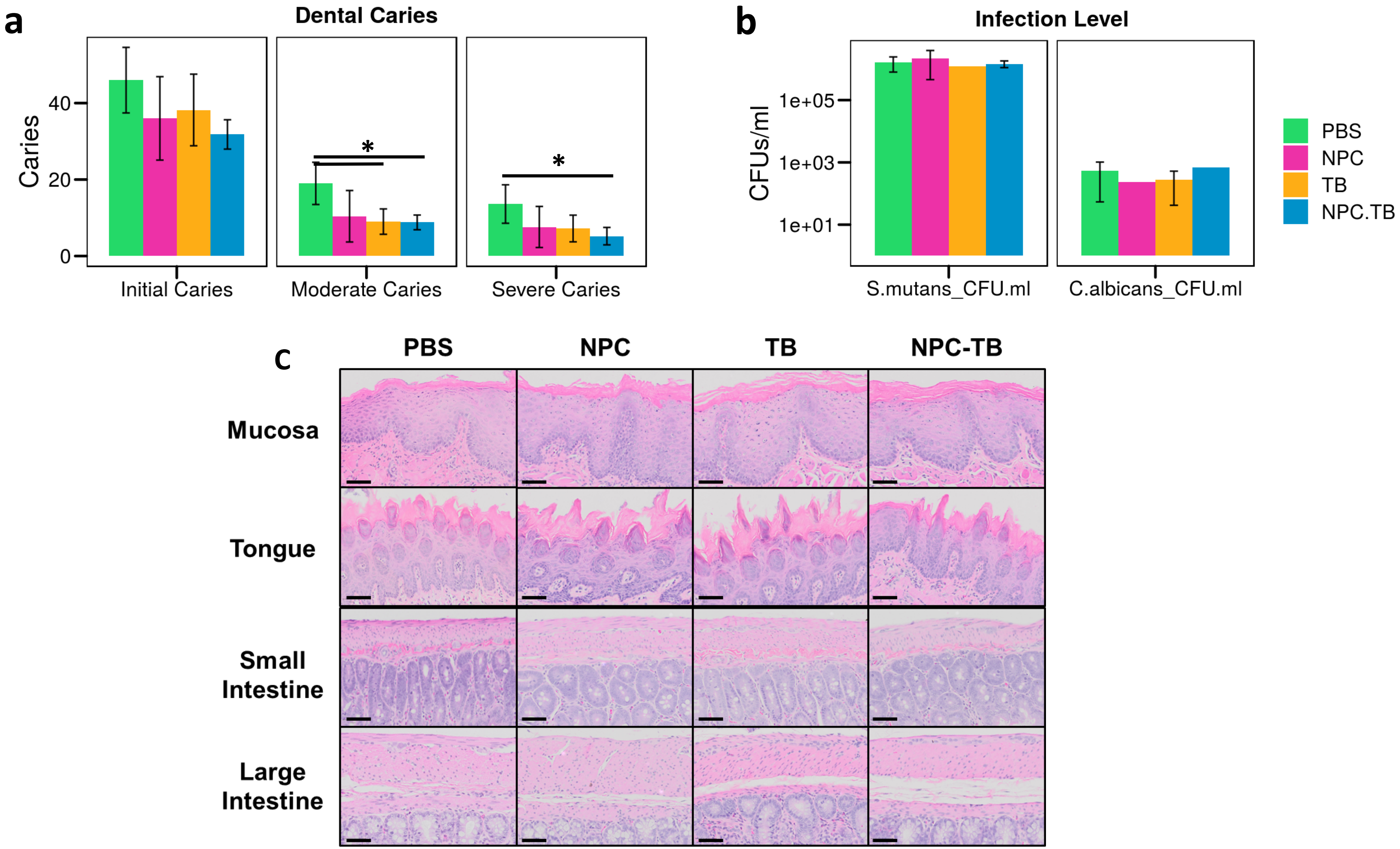


**Supplementary Figure 1. Clinical characteristics of animal model after treatment.** (a) Dental caries bar plot indicates the number tooth surface lesions. In our model, tooth enamel progressively develops caries lesions (analogous to those observed in humans), proceeding from initial areas of demineralization to superficial, moderate, and on to extensive (severe) lesions characterized by enamel structure damage and cavitation. Groups are separated by severity of the lesion from initial to severely affected dentin using Larson's modification of Keyes' scoring system. (b) Infection level bar plots indicates viable cells (CFU) of *S. mutans* and *C. albicans* recovered from dental plaque using selective culture media Mitis Salivarius Agar with bacitracin and ChromAgar, respectively. (c) Histological images of the oral mucosa, tongue, and gastrointestinal tissues following treatment with PBS, NPC, TB, and NPC.TB using Hematoxylin and Eosin staining. Scale bar = 50 µm. Images were taken using 40x magnification. * = $P < 0.05$, Wilcoxon test.

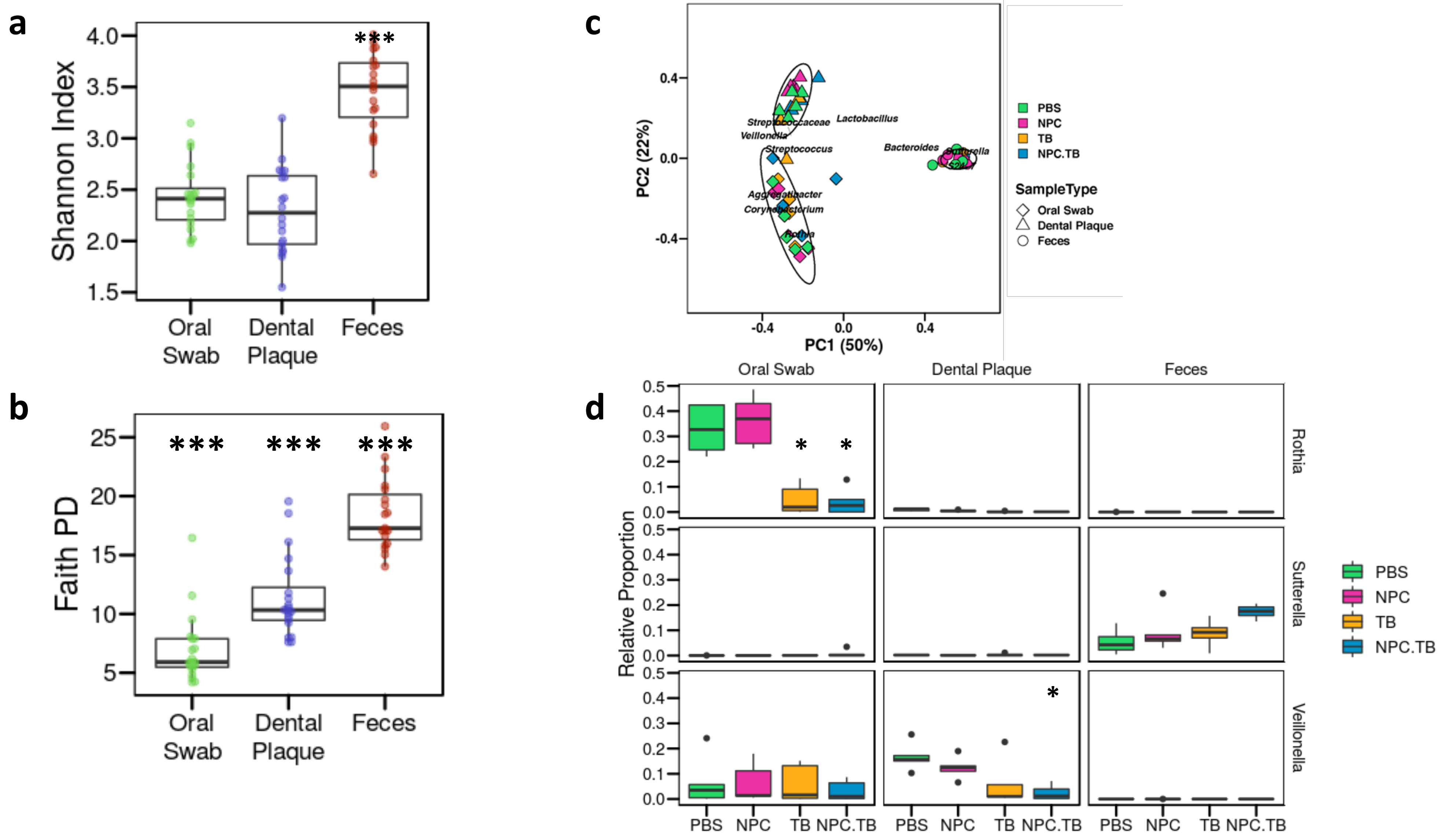


**Supplementary Figure 2. Bacterial taxa and diversity differences among groups.** (a) Shannon index characterizing bacterial diversity within each body site. Gut site (feces samples) sample types were more diverse compared to oral sample types. (b) Phylogenetic diversity index used to identify the diversity is distributed across different branches of the tree of life. All the groups were statistically significant different with a range from 5 to 18 times more diverse ASV species for oral versus gut sites. (c) Beta-diversity principal coordinate analysis using Bray-Curtis distances indicating sample distribution by the microbiome profiles. Color code was used for treatment groups and shapes for oral-gut sample body sites. (d) Main bacterial taxa among treatment groups (pairwise comparison control groups PBS and NPC against TB-containing groups). Asterisk: * = $P < 0.05$; *** = $P < 0.001$, Wilcoxon test.

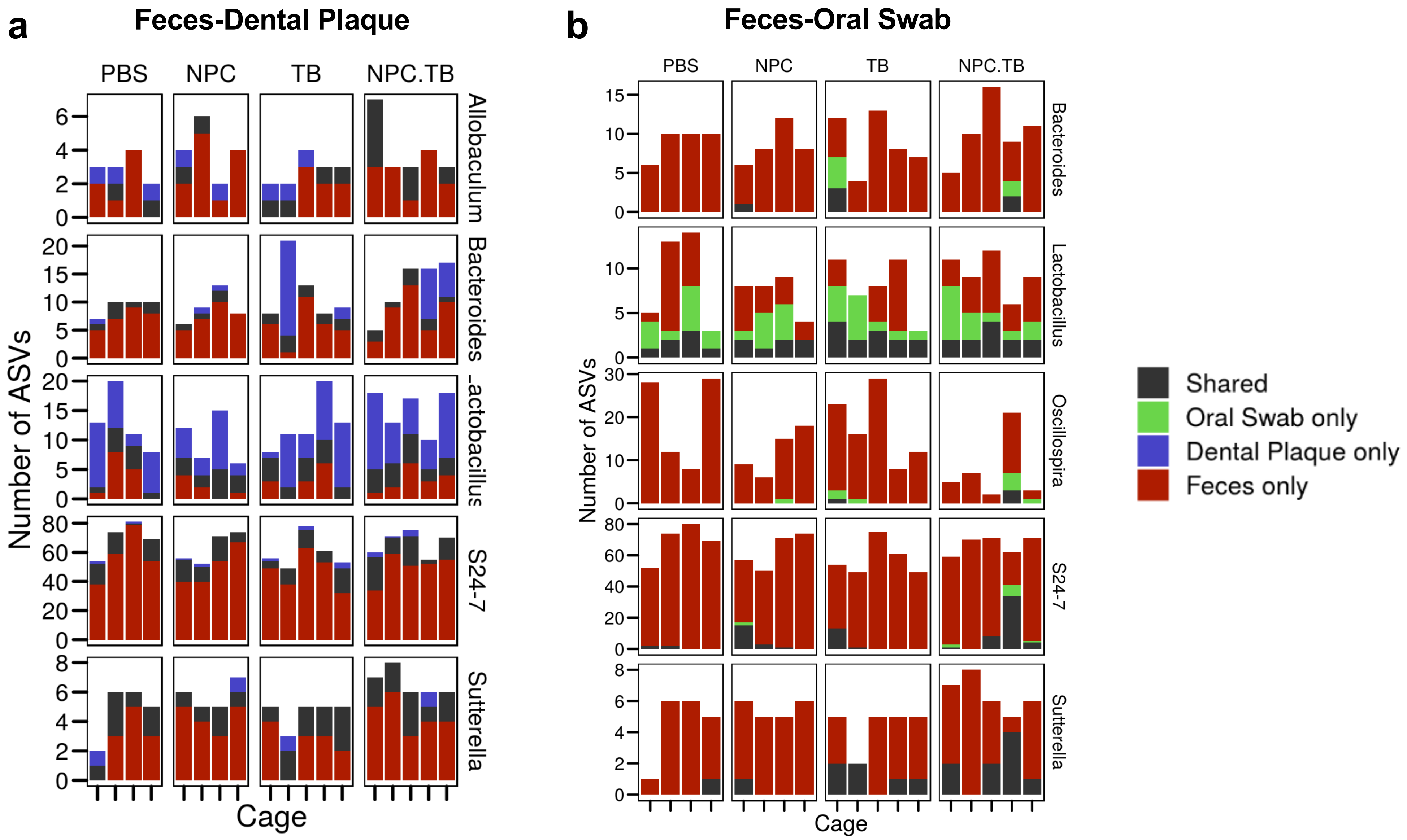


**Supplementary Figure 3. Shared ASV in gastrointestinal sites in animal model using linear model.** Main bacterial taxa found in treatment groups associated with the fraction of oral swab, dental plaque, and fecal amplicon sequence variant (ASVs) appearing shared (both) and unshared (only) between body sites

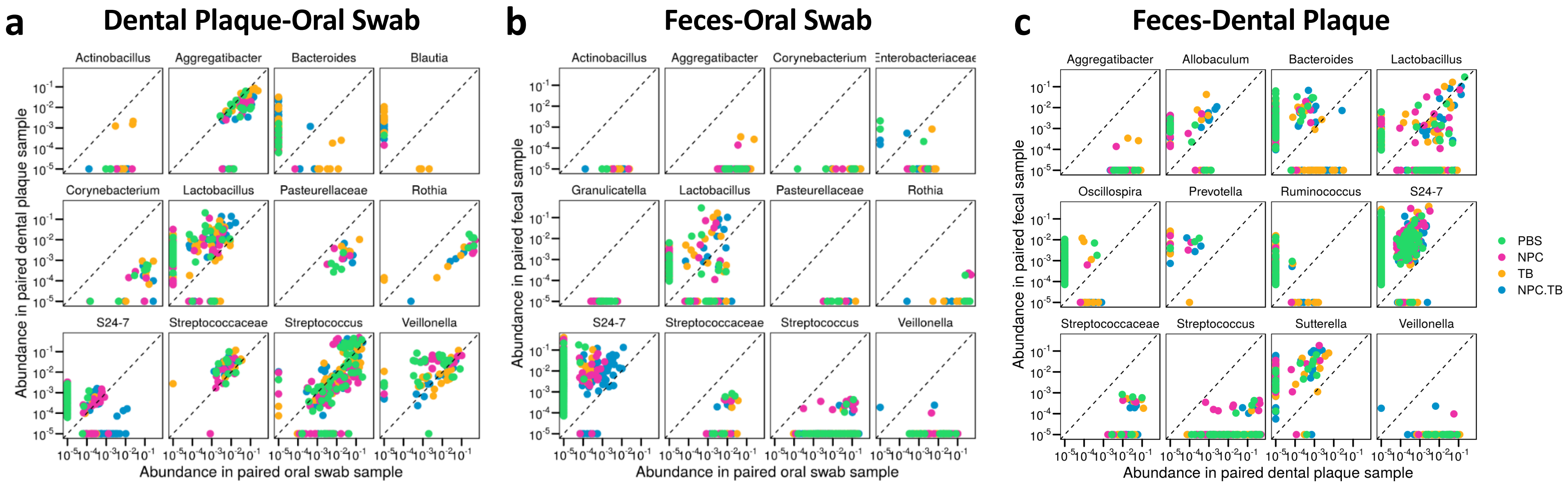


**Supplementary Figure 4. Gastrointestinal sites nestedness pairwise for the 12 main bacterial genera.** Microbial taxa associated paired among body sites using the relative abundance for each animal included in the study. The panels are divided in 3 comparisons including all the permutation between body sites. The intercept line in each graph indicate shared area in the pairwise comparison for each bacterial taxon. Points correspond to the individual amplicon sequence variant (ASV) identified in each bacterial genus. Color code used corresponds to treatment groups included in this study.
